# Structural Equation Model of Elementary School Students’ Quality of Life Related to Smart Devices Usage Based on PRECEDE Model

**DOI:** 10.3390/ijerph18084301

**Published:** 2021-04-18

**Authors:** Jin-Pyo Lee, Yang-Sook Lee

**Affiliations:** 1 Department of Nursing Science, U1 University, Yeongdong-gun 29131, Korea; jplee@u1.ac.kr; 2 Department of Nursing Science, Kongju National University, Gongju 32588, Korea

**Keywords:** quality of life, smart devices addiction, elementary school students, health-promoting behavior, family environment, self-efficacy, social support, smart device related parental intervention

## Abstract

Korean elementary school students have the lowest life satisfaction levels among OECD countries. The use of smart devices has led to smartphone addiction, which seriously affects their quality of life. This study aims to establish and test variables that affect the quality of life (QOL) of elementary school students based on the Predisposing, Reinforcing and Enabling Constructs in Educational Diagnosis and Evaluation (PRECEDE) model, using smart device-related parental intervention, self-efficacy, social support, health promotion behaviors, family environment, smart device addiction, and QOL as measurement variables. Three elementary schools in the Republic of Korea completed self-report questionnaires. Descriptive statistical analysis and hypothetical model fit and test were used for data analysis. The model was found to be valid. Smart device addiction directly affected QOL. In contrast, health promotion behaviors, self-efficacy, social support, and smart device parental intervention indirectly affected QOL. Health-promoting behaviors also directly affected smart device addiction, self-efficacy, and family environment had a direct effect on health-promoting behavior. Therefore, to improve the QOL of elementary school students, the government should focus on developing programs that can help them actively perform health promotion activities and improve self-efficacy, social support, and parental intervention for smart devices that indirectly affect them.

## 1. Introduction

The life satisfaction level experienced by elementary school students in Korea was the lowest among 30 OECD countries in 2013 [1]. Korean children’s satisfaction with life in 2018 was 6.6, which is lower than the average score of 7.6 of 27 OECD and European countries [2]. Elementary school students in Korea are generally not satisfied with their lives, and their quality of life (QOL) has deteriorated. QOL can be expressed as satisfaction, happiness, and subjective well-being, and is influenced by various variables such as individual physical function, psychological state, and social interactions [3].

Among elementary school students from the fourth to the sixth grade in Korea, the smartphone ownership rate is 74.2% [2], and among elementary school students, smartphones have been established as a tool for communicating with their peers and playing culture. With the development of a model scientific civilization, smart devices have become a practical tool for elementary school students as a means of communicating with their peers, and meets their desire to build closer friendships. For them, the playground is no longer a realistic place to play freely. Given the ever-expanding culture of media play centered on smart devices, the problem of smartphone addiction is a sociopathic phenomenon that seriously affects the QOL of elementary school students [4].

Smart devices are multimedia devices that emphasize portability with smartphones, tablet PCs, and other electronic devices that can be operated with fingers or touch pens [5]. Smart phones are the smart devices most familiar to elementary school students. The smart device-related problem most associated with elementary school students is smartphone addiction [6]. Negative psychological symptoms such as depression, a general tendency to avoid interpersonal relationships, and impulse control disorders, among others caused by the excessive use of smart devices, and problem behavior in daily life such as academic non-adaptation and speech destruction can be combined, leading to poor QOL [7,8,9].

According to the National Survey on Internet and Smartphone Usage of 2018, smartphone dependency is increasing in the first to third grades of elementary school [10]. In addition, elementary school students are inadvertently exposed to inappropriate media advertisements during the use of smart devices, resulting in a vicious cycle of increased use of social networking sites (SNS) and the Internet for students who are unhappy with their lives. Further, the higher the level of addiction to smart devices, the lower the QOL [11,12,13]. Therefore, it is necessary to identify the relationship between elementary school students’ use of smart devices and their QOL.

One of the key variables involved in the use of smart devices by elementary school students is health promotion. Health promotion behavior refers to a multi-dimensional pattern in which self-realization, the individual’s level of well-being, and satisfaction are maintained and strengthened by habitual behavior regardless of the disease [14]. Health promotion activities include increasing physical activity and curbing elementary school students’ use of smartphones. As a result of mediating physical activity in elementary school students, smartphone game time has significantly decreased [15]. Thus, the more serious the game addiction, the better it is to perform health promotion activities [16].

Positive parenting of children at home is the best way to prevent smart device addiction [17]. Therefore, parents should recognize the importance of intervening in the use of smart devices by elementary school students. However, parents are not good role models for using smart devices and lack awareness of how to discipline such use [18]. Smart device parent intervention indicates the interaction between parents and children related to smart devices, in particular, the parents’ direct or indirect involvement in the child’s media use or attitude [19]. Support from family members, especially parents, significantly influences the personality, character development, and behavior of elementary school students. Elementary school students who feel a lack of emotional support and understanding from their parents tend to fulfill their desire for understanding and attention through Internet cyberspace [20]. When elementary school students talk to their parents often, learn how to use smart devices from their parents, and feel positively supported by their parents, their internet addiction scores are low and their self-efficacy is high [21].

Self-efficacy is the judgment and belief in one’s ability to overcome a situation on one’s own and to execute a task entrusted to them successfully [22]. It has a partial mediating effect on smartphone overuse: when self-efficacy increases, smartphone overuse is lowered [23]. Young’s (1999) study found that Internet addiction was frequent in people who lacked socially supportive experiences [24]. In addition, those who lack practical close relationships due to the development of the Internet and SNS are still willing to satisfy their desire to be supported in the virtual world [13]. Those who feel a lack of social support are more likely to become addicted to smart devices because of the use of smart devices and its excessive digital content [25], among other factors related to the use of smart devices that negatively affect the QOL of elementary school students.

Green and Kreuter (2005) proposed the Predisposing, Reinforcing and Enabling Constructs in Educational Diagnosis and Evaluation (PRECEDE) model which evaluates several stages of diagnosis to understand health-related factors on QOL. The PRECEDE model has been used to plan and assess health promotion programs to address health issues [26]. To date, the PRECEDE model has been studied using the concepts presented in the model in whole or in part according to the health problems of various subjects, but research on smart device problems in elementary school students is lacking. For this reason, it is necessary to apply the factors affecting the QOL of elementary school students in the use of smart devices in the PRECEDE model.

The current study established and verified a structural equation model (SEM) based on the PRECEDE model by selecting smart device addiction, health promotion activities, home environment, self-efficacy, social support, and smart device parent arbitration as factors affecting the QOL of elementary school students using smart devices. We further identified causality and influence through each step of the diagnosis.

This study aimed to identify factors affecting the QOL of elementary school students in relation to their use of smart devices, by applying the PRECEDE model, and to establish a hypothesis model to verify the validity of the model.

Specific research objectives were as follows.

(1) Suggest a hypothetical model that explains the QOL of elementary school students using smart devices based on the theoretical background.
(2) Test the suitability between the hypothetical model and the actual data and constructs and verify the structural model of the variable relationship.
(3) Identify direct and indirect effects among variables affecting QOL and establish their mutual causal relationships.

## 2. Materials and Methods

### 2.1. Study Design

**Figure 1:**
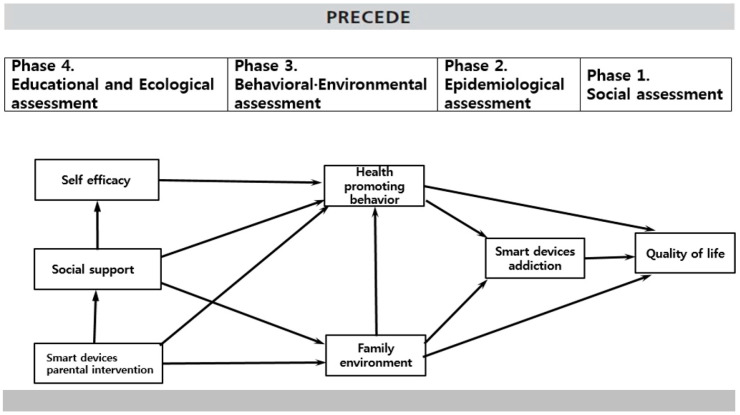
Concept of the study applying the PRECEDE model.

Based on the PRECEDE theory of Green and Kreuter (2005), this study identified factors affecting the QOL of elementary school students using smart devices through a literature review and established a theoretical framework (Figure 1). Specifically, QOL was set as the dependent variable, and smart device addiction was set as a health problem for those whose QOL was affected. The higher the smartphone usage in elementary school students, the more the QOL was negative [11]. Therefore, paths were set, including factors that affect the QOL in relation to the addiction of smart devices for elementary school students [7,8]. Smartphone addiction is a type of technological addiction that affects QOL [8]. As the age of smart device users is gradually decreasing and smart devices are more commonly used in elementary school students’ daily lives [1], they are more susceptible to smart-device addiction, a health issue that affects their QOL.

Health promotion behavior was selected as a behavioral factor, and environmental factors that affected smart device addiction, and attachment and communication between parents and children were included as family environmental factors. Prior studies have shown that the more the Internet game addiction is severe, the less likely people are to engage in health promotion activities [16,27]. For elementary school students, family environment is important. A prior study indicated that if the relationship between parents and children is not amicable, the less frequently they communicate with each other, the more likely they are to use smartphones [7,28]. Based on the results of prior studies, this study selected two factors: behavioral factors and family environment factors. Specifically, we explored health promotion as the behavioral factor affecting elementary school students’ smart device addiction and communication between parents and children and attachment between parents and children as family environment factors.

Self-efficacy was set as a predisposing factor, social support as a reinforcing factor, and smart device parent arbitration as an enabling factor. The lower the self-efficacy, the higher the degree of Internet addiction [22,29]. If self-efficacy is high, unhealthy habits associated with Internet use can be controlled by themselves [20]. Self-efficacy is also a factor affecting health promotion activities and is a powerful predictor of health promotion activities [30,31]. Low social support is a predictor of smart device addiction [32]. The extent to which parents are aware of the risks associated with smart device addiction and parental influence are related to their children’s smart device addiction [7]. Parents play an important role in guiding, managing, and mediating children’s desirable use of smart devices [33]. Parents’ attitudes and roles in using smart devices in their homes have a significant impact on their children’s use and the habit of smart devices because they teach them how to use it [28,33]. Therefore, smart device parent intervention was established as a social and environmental resource to help elementary school students perform actions that are conducive to their health.

Based on the theoretical framework of this study and the results of prior studies, the paths between the variables were determined, and a hypothesis model was established, as shown in Figure 1.

### 2.2. Research Design

This structural model study built a hypothesis model based on the PRECEDE model to identify factors affecting the QOL of elementary school students on the use of smart devices and verified the suitability of the model and the hypotheses presented in the model through the collected data.

### 2.3. Participants

The participants included fourth, fifth, and sixth graders from elementary schools in Daejeon and Sejong city, who gave their written consent to participate in the study. A sample size of 200 was recommended for the maximum likelihood estimation (MSE) most used in structural equations, with 10 to 20 times the observed variable allowance being recommended for the number of samples required [34]. This study collected 246 questionnaires, of these 23 were excluded because they were inaccurate, and inconsistent for coding purposes, so finally 223 copies were analyzed.

### 2.4. Research Instrument

The general characteristics of the respondents included gender, grade, daily time use of smart devices, and frequency of use. To ensure the validity of the instruments used in this study, confirmatory factory analysis (CFA) was conducted.

#### 2.4.1. Self-Efficacy

Based on Bandura’s theory, the self-efficacy instrument developed by Sherher and Mddux [35] was translated and used by Jeong [36] to fit elementary school students. This tool consisted of 18 questions with a total of three sub-constructs of confidence, self-regulated efficacy, persistence, and each question used a 5-point Likert scale (1 = “not at all”, 5 = “very much so”). A higher score indicates a higher degree of self-efficacy. In this study, Cronbach’s alpha was 0.94.

#### 2.4.2. Social Support

The social support instrument developed by Dubow and Ullman [37] was modified by Jeon [38]. This instrument consisted of a total of ten questions in the sub-construct of instrumental support, appraisal support, and emotional support, each of which was evaluated on a Likert scale (1 = “not at all”, 5 = “very much so”). The higher the score, the higher the level of social support. In this study, Cronbach’s alpha was 0.94.

#### 2.4.3. Smart Device Parental Intervention

Smart device parental intervention was measured by an instrument that Park [39] modified based on the tool developed by Nathanson [40]. This instrument consisted of a total of 14 questions in the sub-construct of positive and negative interventions, each of which was evaluated on a five-point Likert scale ranging 1 (“not at all”) to 5 (very much so). Cronbach’s alpha in this study was 0.70.

#### 2.4.4. Health-Promoting Behavior

This study used a health promoting behavior tool that No [41] constructed and Im and Jeong [42] modified. The instrument in this study consisted of a total of 27 questions, including personal hygiene, eating habits, exercise, health interest, mental health, among others, evaluated on a four-point Likert scale ranging from 1 (“not at all”) to 4 points (“always do so”). The higher the score, the higher the degree of health promotion. In this study, Cronbach’s alpha was 0.84.

#### 2.4.5. Family Environment

Parent–child communication: This study used the parent–child communication instrument developed by the Korean Institute of Criminology [43] and modified by Kim [44]. It includes a total of four questions, which are evaluated on a five-point Likert scale ranging from 1 (“not at all”) to 5 (“very much so”). The higher the score, the better the communication between parents and children to promote mutual understanding. In this study, the Cronbach’s alpha was 0.85.

Parent–child attachment: This study used the parent–child attachment instrument developed by Riner [45] and translated and modified by Kim [44]. The instrument consisted of a total of four questions evaluated on a five-point Likert scale ranging from 1 (“not at all”) to 5 (“very much so”). In this study, Cronbach’s alpha was 0.84.

#### 2.4.6. Smart Device Addiction

This study used the smart device addiction instrument that Jeong [5] modified for smart devices, based on the Internet addiction instrument developed by Young [24]. The instrument consisted of a total of 13 questions evaluated on a five-point Likert scale ranging from 1 (“not at all”) to 5 (“very much so”). In this study, Cronbach’s alpha was 0.91.

#### 2.4.7. Quality of Life

This study used a youth self-reported instrument for the PedsQLTM 4.0 Generic score scale developed by Varni [46] and validated by Choi [47]. The instrument was developed to assess the cognitive development of elementary school students, and the sub-construct of QOL consisted of physical, emotional, social, and academic areas. It consisted of 23 five-point Likert scale items ranging from 1 (“no problem at all”) to 5 (“almost always a problem”).

### 2.5. Data Collection and Ethical Considerations

Data collection was conducted by one researcher and two research assistants from July to September 2018. Elementary schools located in Deajeon city and Sejong city received written consent from their guardians, explained the purpose of this study to the subjects, and conducted surveys to students who agreed to participate in the study. The survey took 30 min to complete, and participants could withdraw from the study at any time. Those who participated in the study received a small compensation.

### 2.6. Data Analysis

The collected data were analyzed at a significance level of 0.05 using SPSS version 23 (IBM, Armonk, NY, USA) and AMOS 21 (IBM, Armonk, NY, USA). The verification of the general characteristics and normality of the subjects were analyzed using descriptive statistics. Correlation and multicollinearity between the variables were analyzed using Pearson’s correlation coefficient, and the verification of the normality of the samples was confirmed using skewness and kurtosis.

Confirmatory factor analysis (CFA) was performed to verify the validity of the instrument by applying ML (perform bootstrap = 500). The fit indices were χ^2^ verification, χ^2^/df, goodness of fit index (GFI ≥ 0.9), root mean square residual (RMR ≤ 0.05), and root mean square error of approximation (RMSEA = 0.05∼1). This study used the following incremental fit indices: normed fit index (NFI ≥ 0.9), incremental fit index (IFI), comparative fit index (CFI ≥ 0.9), and Tucker–Lewis index (TLI ≥ 0.9). Moreover, the parsimonious fit index was analyzed using the adjusted goodness of fit index (AGFI ≥ 0.9) and the parsimonious normalized fit index.

The significance test of the paths of the hypothesized model was confirmed by the standardized regression weight, critical ratio, *p* values, and the explanatory power of the endogenous variables was confirmed by the estimate of squared multiple correlation (SMC). Bootstrapping (=500) was used to test the statistical significance of the total, direct, and indirect effects of the hypothesis model.

## 3. Results

### 3.1. Quality of Life by General Characteristics

Table 1 shows differences in the quality of life by general characteristics. Participants comprised 117 male students (52.5 %) and 106 female students (47.5%). By grade, there were 76 fourth grade students (34.1%), 70 fifth grade students (31.4%), and 77 sixth grade students (34.5%). Eighty-four students (37.7%) responded that they used smart devices for one to three hours a day. Regarding how often smart devices were used, 146 students (65.6%) used them six to seven times a week. The difference in QOL according to the daily use time and frequency of smart devices was significant (F = 8.851, *p* < 0.001; F = 5.554, *p* < 0.001). Specifically, the group that used smart devices for 10 to 30 min a day had a higher QOL than the other group.

### 3.2. Descriptive Statistics, Normality, and Multicollinearity Tests of Variables.

The mean scores for smart device parent intervention, social support, and self-efficacy were 2.57, 4.10, and 3.63, respectively. In addition, the mean scores of respondents’ health promotion, smart device addition, and QOL were 3.10, 2.30, and 4.36, respectively. In this study, the absolute values of skewness and kurtosis were less than 2 and 7, respectively, indicating that they were normally distributed. In addition, the correlation among the study variables were all less than 0.70, the tolerance limit was greater than or equal to 0.10, and the variance inflation factor (VIF) was less than 10; therefore, it was confirmed that no problem of collinearity was found among the study variables (Table 2).

### 3.3. Test of Structural Model

#### 3.3.1. Validity of the Study Variable

This study identified convergent validity and discriminant validity to determine whether the construct concept was accurately measured by the variables. As a result of checking the average variance extracted (AVE) and reliability, AVE was found to have met the score of 0.5 or higher, and concept reliability was more than 0.7, making it more relevant. The mean variance extraction (AVE: 0.541–0.977) was found to be greater than the square of the correlation coefficient value (0.0002–0.456) among the latent variables, and the discriminant validity was satisfied.

#### 3.3.2. Fitness Statistics of the Hypothetical Model

As a result of the fitness test of the hypothetical model, χ^2^/df was 2.48, fit below 3, and other indices (GFI = 0.90, RMR = 0.05, RMSEA = 0.08) met the criteria. Incremental fit indices (NFI = 0.88, IFI = 0.93, CFI = 0.93, TLI = 0.90) were appropriate. The AGFI, which is distributed between 0 and 1, was 0.84 and PNFI was 0.68, indicating that PNFI was greater than 0.6. Overall, the goodness of fit index met the criteria and was determined as the final model because it was assessed to be parsimonious.

### 3.4. Parametric Estimation and Effect Analysis of Hypothetical Model

**Figure 2:**
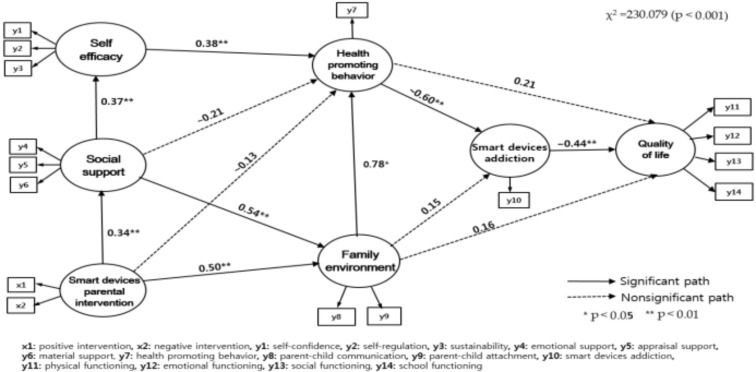
Path diagram of the hypothetical model.

As a result of analyzing the hypothetical model of this study, eight of the 13 paths were statistically significant. The coefficients and significance test results for each path are shown in Figure 2 and Table 3.

The variable that affects social support is smart device parent intervention (γ = 0.34, *p* = 0.002) with 11.3% explanatory power. The variable that affects self-efficacy was social support, with 13.5% explanatory power (β = 0.37, *p* < 0.001). The variables that affect the family environment are social support (β = 0.54, *p* < 0.001) and smart device parent intervention (γ = 0.50, *p* < 0.001), which have 72.3% explanatory ability power to describe the family environment. Variables affecting health-promoting behaviors were self-efficacy (β = 0.38, *p* < 0.001) and family environment (β = 0.78, *p* = 0.012), with a 54.9% explanatory power to describe health-promoting behaviors and 26.5% explained. The variables that significantly affected the QOL were smart device addiction (β = −0.60, *p* < 0.001), which had a 43.1% explanatory power for the QOL.

The results of the analyses of the direct, indirect, and total effects of the hypothesis model were as follows: smart device parent intervention (γ = 0.34, *p* = 0.002) had a significant direct effect on social support, and social support (β = 0.37, *p* < 0.001) had a significant direct effects on self-efficacy. Self-efficacy (β = 0.38, *p* < 0.001) and family environment (β = 0.78, *p* = 0.012) had direct effects on health-promoting behaviors. Social support (β = 0.56, *p =* 0.011) and smart device parent intervention (β = 0.51, *p =* 0.024) had indirect effects on health-promoting behaviors. Social support (β = 0.54, *p* < 0.001) and smart device parent intervention (γ = 0.50, *p* < 0.001) had direct effects on family environment.

Health-promoting behaviors for smart device addiction had a direct effect (β = −0.60, *p* < 0.001). Family environment (β = −0.46, *p* = 0.030), self-efficacy (β = −0.23, *p* = 0.010), and social support (β = −0.13, *p* = 0.020) had indirect effects on smart device addiction. Smart device addiction (β = −0.44, *p* < 0.001) had a direct effect on QOL, and health-promoting behaviors (β = 0.27, *p* = 0.010), self-efficacy (β = 0.18, *p* = 0.010), social support (β = 0.22, *p* = 0.010), and smart device parent intervention (β = 0.24, *p* = 0.029) had indirect effects on QOL.

## 4. Discussion

This study aimed to establish and validate a structural model for elementary school students on the use of smart devices as basic data for strategies to improve their QOL, based on the PRECEDE model.

Among the factors affecting QOL identified in the first stage of the social assessment, addiction to smart devices had a direct effect, accounting for 43.1% of the QOL. In prior studies, the higher the use of smart devices, the more negatively it affected QOL [11,48]. This reflects studies showing that the higher the addiction to smart devices, the lower the subjective happiness index [12]. In addition, a study on the relationship between Internet and SNS use and QOL, suggested that the Internet was used as an alternative means of happiness. In particular, the higher the frequency of SNS use, the greater the stress, the lower the satisfaction of the subjects’ lives [13]. This combination of negative psychological symptoms and problem behavior in everyday life is caused by smart device addiction, thereby reducing the QOL [6,7]. Moreover, it can negatively impact the physical and mental health of elementary school students who are not yet mature in self-control or judgment, if they use smart devices indiscriminately, which in turn can affect their QOL. Therefore, guiding the right use of the smart device and monitor its use continuously is necessary to improve elementary school students’ QOL. Meanwhile, smart device parent intervention, social support, self-efficacy, and health-promoting behaviors had indirect effects on QOL. The more positive the smart device parent intervention, the higher the social support, and the higher the self-efficacy, the better QOL for elementary school students. The above results suggest that, to improve the QOL of elementary school students, it is necessary to examine the degree of addiction to smart devices and develop diverse strategies to strengthen various factors that have indirect effects.

In the second stage of the epidemiological assessment, health-promoting behaviors were identified as variables that directly affected addiction to smart devices. Increased health-promoting behaviors such as physical activities reduced smartphone addiction in elementary students [15]. In addition, students with higher levels of Internet addiction had difficulties in self-regulation, which reduced their health-promoting behaviors [16]. Consequently, as the use time of smart devices increases, daily life becomes boring due to the exposure of provocative media, which reduces health-promoting activities. Therefore, to reduce the addiction to smart devices in elementary school students, programs that can increase health-promoting activities need to be developed. In this study, self-efficacy, social support, and family environment were identified as variables that had indirect effects on smart device addiction. A prior study, in which self-efficacy was partially mediated by smartphone addiction, supported the results of this study [23]. Those who were reluctant or uncomfortable to get close to others in real life had a higher rate of SNS use and sought to satisfy the social support they did not get in real life through the virtual world [32]. In this regard, several prior studies have shown that the less intimate the person is with others, the less self-efficacy they possessed and the higher the level of Internet addiction [21,23,29]. Furthermore, the relationship between family environment and smart device addiction showed that parents’ parenting attitudes were negative and coercive, and smartphone addiction was high when parents and children lacked dialog [17,21]. In addition, the Delphi study on smart device addiction said that the problem is not the digital information gap between parents and children, but the inappropriateness of parent discipline and education on smart device use [18]. In this study, the family environment mediated health-promoting behaviors and affected smart device addiction. This means that the closer the relationship with parents and the more frequent the communication, the more children’s health-promoting behaviors increase, which lowers health risks such as smart device addiction [49,50]. Therefore, to reduce the level of addiction to smart devices in elementary school students, it is necessary to help them conduct health-promoting behaviors and develop community-oriented programs that increase their self-efficacy and secure positive support systems in families and society.

Self-efficacy and family environment were the factors that directly affected health-promoting behaviors in the third stage of the behavioral and environmental assessment. Self-efficacy is a significant factor in health-promoting behaviors and supports the results of this study [30,31]. Self-efficacy is the most powerful variable in predicting health-promoting behaviors and can be an important factor in health-promoting behaviors [30,31]. Moreover, the main factor affecting health-promoting behaviors was the home environment. The health-promoting behaviors of elementary school students are greatly influenced by parental guidance, and the role of parents is important [49]. A prior study on parenting and elementary school students’ health risk behaviors indicated that the more receptive and democratic the parenting attitude, the lower the level of health risk behaviors such as drug addiction and smoking [50]. The variables that increased the health-promoting behaviors of elementary school students were self-efficacy and family environment. Therefore, parents need to guide elementary school students so that they can practice health promotion activities, and their self-efficacy needs to be assessed and managed at school and at home. In this study, smart device parent intervention and social support indirectly affected health-promoting behaviors. This means that when there is a smart device parent intervention, social support and self-efficacy is higher, which leads to improved health-promoting behaviors. These results reflected those of studies showing that positive relationships between parents and children increase health promotion behavior through parameters called self-efficacy [51]. This indicates that smart device parent intervention is also included in the parent’s guidance, affecting the child’s self-efficacy, and consequently related to other practices of health-promoting behaviors [51]. Therefore, it suggests that elementary school students need educational programs that can strengthen their self-efficacy and improve their parents’ guidance to practice health-promoting behaviors. According to the environmental assessment, the factors that directly affected the family environment were smart device parent interventions. Prior research has indicated that smart device parent intervention is a necessary resource to reduce elementary school students’ smart device addiction and, thus it is an enabling factor [19]. Therefore, the more positive the parent intervention is concerning smart devices, the stronger the communication and attachment between parents and children [19,28,33]. In addition, the results of this study showed that social support is partially related to the relationship in which the smart device parent intervention affects the family environment. This indicates that if parents properly mediate the use of smart devices and have high social support, a positive relationship between parents and children is formed at home. Thus, parents need to provide appropriate advice and support their children in their use of smart devices.

The results of this study showed that smart device parent intervention influenced social support, while social support affected self-efficacy in the fourth stage of educational and ecological assessment. Moreover, self-efficacy affected health-promoting behaviors, and social support was found to indirectly affect health-promoting behaviors through self-efficacy. Self-efficacy has been identified as a factor that completely mediates social support in influencing health-promoting behaviors. This may be because the higher the social support, the higher the self-efficacy, which increases health-promoting behaviors. Therefore, social support does not increase health-promoting behaviors. Social support also affects self-efficacy, and thus affects the practice of health behaviors [52]. This can be interpreted as a more proactive response to stress when social support is high, leading to a belief in one’s ability to promote self-efficacy and affect the practice of health promotion activities [52].

The result of testing the hypothetical model of the QOL of elementary school students on the use of smart devices, showed that health-promoting behaviors had the greatest influence on smart device addiction. Thus, strategies to activate health-promoting behaviors are required first to reduce addiction to smart devices. A community-centered intervention strategy is needed to develop and expand family support programs that can increase self-efficacy and positively affect the relationship between parents and children. Educational programs for parents on children’s proper use of smart devices should be implemented. Further, a social environment that supports elementary school students needs to be created.

The limitations of this study are the self-reporting instruments; hence, errors cannot be excluded, as elementary school students may respond differently depending on their subjective interpretation of the questions. This study is meaningful in that it is a multidimensional study that shows the factors influencing the QOL of elementary school students who use smart devices, which has been rarely attempted. Based on this study, if research on QOL factors related to the use of smart devices is conducted on adolescents and adults, it will form a meaningful basis for health education and the development of intervention programs.

## 5. Conclusions

Based on the PRECEDE model used in this study, the factors influencing the quality of life of elementary school students on the use of smart devices were constructed and tested step by step. In other words, models were built and verified at each stage, focusing on self-efficacy (predisposing factors), social support (reinforcing factors), parent intervention of smart devices (enabling factors), and family environment and health promotion behaviors (behavioral·environmental factors) that affect the quality of life of elementary school students using smart devices.

The variables that had the greatest influence on the quality of life of elementary school students in relation to the use of smart devices were smart device addiction and health-promoting behaviors. Self-efficacy, social support, and smart devices parent intervention indirectly affected their QOL. Health promotion behaviors affected smart device addiction, self-efficacy and family environment affected health-promoting behaviors, and social support and smart device parent intervention affected the family environment. Based on these variables, we actively encourage developing strategies to improve the quality of life of elementary school students who use smart devices.

This study could be used as a basis for the practical usefulness of elementary school students’ QOL concepts, as it has identified the factors affecting elementary school students’ QOL using smart devices in a multi-dimensional manner, by applying the PRECEDE model. To improve the quality of life for elementary school students, schools and families are recommended to provide counseling by assessing the degree of addiction to smart devices and to apply education mediation programs at practical sites that increase health promotion activities. Furthermore, the PRECEDE model needs to be retested through repeated studies involving various variables related to the use of smart devices for elementary school students.

## Figures and Tables

**Table 1:** Differences in quality of life by general characteristics (n = 223).

Characteristics		N	%	Quality of Life	t/F	*p*	Scheffe (Post Hoc Test)
				M ± SD			
Gender	Male	117	52.5	4.37 ± 0.55	0.362	0.718	
	Female	106	47.5	4.34 ± 0.54			
Grade	Fourth	76	34.1	4.35 ± 0.59	1.647	0.195	
	Fifth	70	31.4	4.45 ± 0.51			
	Sixth	77	34.5	4.28 ± 0.52			
Daily use time	10–30 min ^a^	32	14.3	4.55 ± 0.60	8.851	<0.001	a > b,c,d
	30 min–1 h ^b^	49	22.0	4.48 ± 0.50			
	1–3 h ^c^	84	37.7	4.41 ± 0.45			
	more than 3 h ^d^	58	26.0	4.07 ± 0.57			
Frequency of use	1–2 times a month	9	4.0	4.20 ± 0.78	5.554	0.001	
	1–2 times a week	18	8.1	4.46 ± 0.59			
	3–5 times a week	50	22.4	4.61 ± 0.40			
	6–7 times a week	146	65.5	4.27 ± 0.54			

Note. M: Mean. SD: standard deviation; ^a–d^ indicates each group compared in Scheffe’s test.

**Table 2:** Descriptive statistics and confirmatory factor analyses of measured variables.

Construct concept	Mean	SD	Range	Skewness	Kurtosis	AVE	CR
Endogenous variable							
Smart devices parental intervention	2.57	0.48	1–4	−0.06	0.34	0.650	0.788
Positive intervention	2.59	0.58	1–4	−0.11	0.15		
Negative intervention	2.60	0.51	1–4	0.17	0.53		
Exogenous variable							
Social support	4.12	0.86	1–5	−0.89	0.09	0.781	0.913
Emotional support	4.26	0.92	1–5	−1.26	1.07		
Appraisal support	4.12	0.98	1–5	−0.98	0.25		
Material support	3.95	0.91	1–5	−0.58	−0.26		
Self-efficacy	3.63	0.67	1–5	0.22	−0.44	0.876	0.955
Self-confidence	3.71	0.69	1–5	0.09	−0.59		
Self-regulation	3.55	0.72	1–5	0.16	−0.02		
Sustainability	3.63	0.79	1–5	−0.01	−0.35		
Health promoting behavior	3.10	0.40	1–4	−0.20	−0.53	0.977	0.977
Family environment	4.04	0.76	1–5	−0.50	−0.48	0.541	0.698
Parent–child communication	3.66	1.03	1–5	−0.35	−0.70		
Parent–child attachment	4.43	0.75	1–5	−1.42	1.67		
Smart devices addiction	2.30	0.82	1–5	0.38	−0.42	0.938	0.938
Quality of life	4.36	0.54	1–5	−0.79	−0.14	0.680	0.894
Physical functioning	4.38	0.63	1–5	−1.31	2.91		
Emotional functioning	4.21	0.84	1–5	−1.17	1.33		
Social functioning	4.49	0.62	1–5	−1.13	0.42		
School functioning	4.33	0.68	1–5	−1.34	2.49		

AVE: average variance extracted; CR: construct reliability; SD: standard deviation.

**Table 3:** Standardized estimates, standardized direct, indirect, and total effects for the hypothetical model (N = 223).

Endogenous Variables	Exogenous Variables	SRW (γ, β)	C.R (*p*)	SMC	Direct Effect (*p*)	Indirect Effect (*p*)	Total Effect (*p*)
Social support	Smart devices parental intervention	0.34	3.11 (0.002)	0.113	0.34 (0.010)	-	0.34 (0.010)
Self-efficacy	Social support	0.37	5.29 (<0.001)	0.135	0.37 (0.010)	-	0.37 (0.010)
	Smart devices parental intervention				-	0.12 (0.010)	0.12 (0.010)
Health promoting behavior	Self-efficacy	0.38	5.82 (<0.001)	0.549	0.38 (0.010)	-	0.38 (0.010)
	Smart devices parental intervention	−0.13	−0.61 (0.544)		−0.13 (0.514)	0.51 (0.024)	0.38 (0.010)
	Social support	−0.21	−1.15 (0.247)		−0.21 (0.368)	0.56 (0.011)	0.35 (0.010)
	Family environment	0.78	2.51 (0.012)		0.78 (0.029)	-	0.78 (0.029)
Family environment	Social support	0.54	5.63 (<0.001)	0.723	0.54 (0.005)	-	0.54 (0.005)
	Smart devices parental intervention	0.50	3.59 (<0.001)		0.50 (0.010)	0.18 (0.010)	0.68 (0.010)
Smart devices addiction	Family environment	0.15	1.43 (0.151)	0.265	0.15 (0.375)	−0.46 (0.030)	−0.31 (0.124)
	Health promoting behavior	−0.60	−6.01 (<0.001)		−0.60 (0.010)	-	−0.60 (0.010)
	Self-efficacy				-	−0.23 (0.010)	−0.23 (0.010)
	Social support				-	−0.13 (0.020)	−0.13 (0.020)
	Smart devices parental intervention				-	−0.13 (0.258)	−0.13 (0.258)
Quality of life	Family environment	0.16	1.52 (0.127)	0.431	0.16 (0.260)	0.30 (0.065)	0.46 (0.027)
	Health promoting behavior	0.21	1.83 (0.066)		0.21 (0.144)	0.27 (0.010)	0.47 (0.010)
	Smart devices addiction	−0.44	−5.10 (<0.001)		−0.44 (0.010)	-	−0.44 (0.010)
	Self-efficacy				-	0.18 (0.010)	0.18 (0.010)
	Social support				-	0.22 (0.010)	0.22 (0.010)
	Smart devices parental intervention				-	0.24 (0.029)	0.24 (0.029)

SRW: standardized regression weight; C.R.: critical ratio; SMC: squared multiple correlation.

## Data Availability

The data are not publicly available due to [restrictions eg privacy or ethical].
